# Clinical outcome of implant removal after fracture healing. Design of a prospective multicentre clinical cohort study

**DOI:** 10.1186/1471-2474-13-147

**Published:** 2012-08-15

**Authors:** Dagmar I Vos, Michael HJ Verhofstad, Beate Hanson, Yolanda van der Graaf, Chris van der Werken

**Affiliations:** 1 Department of Surgery, Amphia Hospital Breda, Breda, the Netherlands; 2 Department of Surgery, St. Elisabeth Hospital, Tilburg, the Netherlands; 3 AO Clinical Investigation and Documentation, Dubendorf, Switzerland; 4 Julius Centre for Health Sciences and Primary Care, University Medical Centre, Utrecht, the Netherlands; 5 Department of Surgery, University Medical Centre, Utrecht, the Netherlands

**Keywords:** Implant removal, Metal implants, Fracture healing, Fracture surgery, Osteosynthesis, Complications, Complaints

## Abstract

**Background:**

The clinical results of removal of metal implants after fracture healing are unknown and the question whether to remove or to leave them in is part of discussion worldwide. We present the design of a prospective clinical multicentre cohort study to determine the main indications for and expectations of implant removal, the influence on complaints, the incidence of surgery related complications and the socio-economic consequences of implant removal.

**Methods/Design:**

In a prospective multicentre clinical cohort study at least 200 patients with a healed fracture after osteosynthesis with a metal implant are included for analyzing the outcome after removal. Six hospitals in the Netherlands are participating. Special questionnaires are designed. The follow up after surgery will be at least six months. The primary endpoint is the incidence of surgery related complications. Secondary endpoints are the influence of removal on preoperative symptoms and complaints and the socio-economic consequences.

**Discussion:**

By performing this study we hope to find profound arguments to remove or not to remove metal implants after fracture healing that can help to develop clear guidelines for daily practice.

**Trial registration:**

NTR1297, http://www.trialregister.nl/trialreg/admin/rctview.asp?TC=1297

## Background

In literature only three cohort studies on the results and complications of implant removal can be found [1-3]. No randomized prospective clinical trials have been published. The results of implant removal are unknown and the question whether to remove or to leave metal implants after fracture healing is part of an ongoing worldwide discussion. Most patients relate complaints and symptoms like pain, swelling and stiffness after their fracture has healed to the presence of the metal implant. The question is if these problems are really due to the implant or exist anyway because of the injury, subsequent surgery and/or healed fracture including scar tissue formation. Other important issues in the discussion about the need for implant removal are surgery related complications, postoperative morbidity, the related medical costs and the possible socio-economic consequences. The purpose of this study is (1) to analyse the incidence of surgery related complications, (2) to determine the main indications for implant removal, (3) to analyse the clinical effect on preoperative symptoms and complaints of the patient related to the implant, (4) to determine if the clinical effect of removal meets the main expectations of the patient and the surgeon and (5) to analyse the socio-economic consequences, like the amount of days of absence from work or school due to implant removal. Finally, we hope to find arguments to create guidelines of conduct for removal of metal implants used for osteosynthesis after the fracture has healed.

## Methods/Design

### Design of study

#### Context

For practical and ethical reasons it is impossible to perform a prospective randomised clinical study comparing two groups of patients; one with and one without implant removal after fracture healing. Symptomatic patients with problems and/or complaints that are possibly related to the presence of a metal implant generally wish its removal anyway and likewise the patient and the surgeon would not be motivated to participate in a randomized trial. Therefore, it was decided to perform a prospective multicentre clinical cohort study in adult patients with healed fractures after osteosynthesis with metal implants. Six hospitals in the Netherlands participate in the study, four community teaching hospitals, one community non-teaching hospital and one university medical centre. The study protocol has been approved by the Dutch Medical Ethical Commission and the local Medical Ethical Commissions of each participating hospital. https://toetsingonline.ccmo.nl/ccmo_search.nsf/Searchform?OpenForm Record number NL15133.008.07, ABR number 15133.

#### Questionnaires

Baseline data are recorded preoperatively both by the surgeon and the patient (e.g. age, sex, co-morbidity, length, weight and smoking habits).

To inventory pre- and postoperative symptoms and problems objectively, special questionnaires have been designed. Since no proper validated questionnaire about implant removal exists, questions have been obtained from the validated and generally accepted ‘Disabilities of the Arm, Shoulder and Hand (DASH) scoring system’ [4], which appears to be also suitable for the lower extremity [5] and the ‘Medical Outcomes Study Short Form-36 (SF-36) scores’ [6]. Using the DASH score, evaluation of activities of daily living, social activities, work activities, symptoms, sleeping and confidence is possible. The SF-36 measures physical functioning, limitations in daily activities as result of physical health problems, pain, general health perceptions, vitality and social functioning. Questions about expectations and satisfaction of the result of the implant removal have been designed for both surgeon and patient.

The surgeon answers the questionnaire at six different time points; starting preoperatively after obtaining the informed consent, immediately after the surgical procedure, two weeks postoperatively, after six weeks, after six months in all patients and after one year in case of plate removal (Table 1). The reason for a follow up of one year in case of plate removal is the presumed higher risk of a refracture. A follow up of six months after nail removal is supposed to be long enough to get a reliable overview of possible postoperative complications.

**Table 1:** Follow up schedule

	**Pre- operative**	**Intra-operative**	**2 weeks**	**6 weeks**	**6 months**	**12 months (plate)**
Questionnaire patient	x			x	x	x
Questionnaire surgeon	x	x	x	x	x	x
Clinical examination	x		x	x	x	x
X-ray	x		x		x	x

Surgery related complications (e.g. haemoraghe, infection, nerve damage, poor cosmetic result and refracture) are defined and recorded during the follow up, Additional file 1. The peroperative questionnaire contains questions about the background and level of experience of the performing surgeon, type of anesthesia, use of a tourniquet and antibiotics, type of incision, ease of finding the implant, ease of removing the implant, use of extra instruments for removal, (time) use of fluoroscopy, peroperative complications, estimated blood loss, duration of the procedure (skin to skin), overall satisfaction of the surgeon and grade of difficulty of the surgical procedure according to the surgeon.

Patients fill in their part of the questionnaire preoperatively during the first intake at the outpatient clinic, six weeks postoperatively, after six months and, in case of plate removal after one year (Table 1). These questionnaires contain general questions on length, weight and smoking habits and specific questions about the complaints related to the implant (e.g. pain, swelling, loss of strength, functional impairment). Complaints are rated on a visual analog Likert-scale from 1 (no) to 5 (extreme). Finally, patients are asked to rate the cosmetic aspects of their scar pre- and postoperatively (after six months) on a visual analog scale from 0 (very ugly) to 10 (very beautiful). Both surgeon and patient are asked about their overall satisfaction of the implant removal, if expectations have become truth and if they would perform or undergo the procedure again and why.

#### Clinical examination

Preoperatively, six weeks and six months after operation the surgeon has to examine the mobility of the joints adjacent to the bone from which the implant is removed using a standardized range-of-motion measurement at the outpatient clinic [7]. Also sensory loss and the appearance of the scar are assessed (Table 1)*.*

#### Radiographs

Standard X-rays are taken preoperatively and two weeks, six months and one year (plate) after removal. They are assessed by the surgeon using a list of standardized questions.

#### Data collection

All questionnaires can be filled out on a paper version, but also a special website is developed. One part of this website is freely accessible and contains general information about the study (http://www.implantremovaltrial.nl). The other part of the website is highly secured and only accessible for the patient, surgeon and principle study investigator. In this way all data is instantly ordered in a database and the participating investigators can monitor the exact follow up.

#### Inclusion criteria

Only adults (≥ 18 years) with a clinically and radiologically healed fracture after a plate- or intramedullary nail osteosynthesis of the upper extremity (radius, ulna, humerus, clavicle) or lower extremity (femur, tibia) with an ASA-classification of I, II or III according to the American Society of Anaesthesiologists [8] and a signed informed consent (after agreement on removal) are included.

#### Exclusion criteria

Excluded are all patients younger than 18 years, ASA-classification IV and V, patients with a nonunion and/or infection, patients with implants in other than above mentioned bones and all patients without informed consent.

#### Patient selection and informed consent

All adult patients (≥ 18 years) in the six participating hospitals with a clinically and radiologically healed fracture of the clavicle, humerus, radius, ulna, femur or tibia (proximal, midshaft or distal), who have been treated with a plate or nail osteosynthesis, are invited to participate in the study. Healed fractures of the hand, acetabulum, spine or feet are left out of the study because of the low incidence. Also any osteosynthesis of the fibula are not encountered in the study because there hardly is any discussion about implant removal of fibula osteosynthesis in case of complaints. We choose to include clinically and/or radiologically healed fractures and not for a minimum time period between the fracture treatment and the implant removal, nor was confirmation of consolidation by a CT-scan required. It was presumed that this will resemble daily clinical practice most reliably. Patients can be symptomatic, but also reasons like ‘*the implant doesn’t belong in my body’* and *‘I want to get it out’* are accepted reasons for implant removal. After a general informed consent on the standard removal procedure by the surgeon and a decision by patient to have the implant removed, the surgeon informs the patient verbally about this study. In case of interest, the patient is subsequently offered a written information letter. After agreement on participation the patient is asked to sign a written informed consent form.

#### Intervention

The surgical procedure itself is performed in a normal daily practice to minimize any form of bias because of the study. This means that the surgery can be performed under general, regional or local anaesthesia. The operating surgeon is a graduated general-, trauma- or orthopaedic surgeon or a resident in training for one of these specialties. The surgeon is free to use fluoroscopy, antibiotics, a tourniquet and thrombosis prophylaxis, according to his local hospital protocol. Also the surgeon decides on the postoperative treatment, like weight bearing and the resumption of work or sport activities. Postoperative pain is treated according to the normal guidelines of the participating hospitals (paracetamol, NSAID’s, morphine).

### Design of data collection

#### Primary end point

The incidence of surgery related complications (i.e. postoperative haemorrhages, wound infection, nerve injury, refracture, poor cosmetic result and general complications)

#### Secondary end points

Any changes (improvement or worsening) in preoperative signs, symptoms and complaints of the patient after implant removal.

The expectations on the outcome of implant removal, for both patient and surgeon, in terms of pain, paresthesia, loss of strength, stiffness, swelling, problems in daily living and cosmetic result.

The resumption of work and sport activities.

### Statistical analyses

#### Sample size calculation

In literature the surgery-related complication rate varies from 3 to 40% [9].

In a cohort of 200 patients about 30 surgery-related complications are foreseen. With this amount of patients an overall complication rate of 15% can be estimated with a 95% confidence interval of 11 to 21%. Factors that increase the complication rate with a relative risk of more than 2 (95%CI 1.0-4.1) can be ascertained with this sample size.

#### Data analysis

Baseline characteristics and the intra-operative data of the patients will be described for each type of implant (clavicle plate, clavicle nail, humerus plate, radius plate, ulna plate, femur plate or nail and tibia plate or nail). Outcome parameters are grouped for the upper extremity (including all upper extremity implants) and lower extremity (including all lower extremity implants). Dropouts and withdrawals will be analysed for the reason of termination.

The number of complications will be calculated. Relations between patient characteristics (e.g. age, sex, comorbidity, use of medication, smoking habits, profession, sport activities), fracture data (e.g. type of fracture, localisation, type of osteosynthesis used, type of material) and complications are estimated with logistic regression and corresponding 95% confidence intervals are calculated.

Distribution measures for the secondary endpoints (changes in complaints and the estimation of both the surgeon and the patient on the outcome of the operation) will be calculated preoperative vs six months postoperative. Visual analog scales (VAS scores) are used pre- and six months postoperatively to measure the amount of pain and the cosmetic result.

In case of continuous outcome variables, a Student *t*-test (paired tests) and in case of categorical outcome variables a chi-square test will be used for statistical analysis. The Wilcoxon signed rank test will be used for paired variables. Because of multiple testing a *p* value < 0.005 is presumed to be significant.

## Discussion

This prospective multicentre clinical cohort study is primarily designed to evaluate the outcome and complications of implant removal after fracture healing.

No literature describing a randomized controlled trial on implant removal is available and to our knowledge there has never been published a design for a study about implant removal before. By performing this study and finally by publishing the outcome more profound arguments can be given whether to remove or not to remove metal implants after fracture healing, with the development of clear guidelines for daily practice.

## Competing interests

This study was made possible due to a research grant by the AO foundation, AO Research Grant S-09-37V (Davos, CH). The authors declare that they have no competing interests.

## Authors’ contributions

DV is the main author of the study design, performance and initiation. MV participated in the design, performance and first initiation of the study. BH contributed to the methodology of the study design. YG contributed to the methodology of the study design and statistical analysis. CW participated in the study design and first initiation of the study. All authors read and approved the final manuscript.

## Pre-publication history

The pre-publication history for this paper can be accessed here:

http://www.biomedcentral.com/1471-2474/13/147/prepub

## Supplementary Material

Additional file 1 Definitions of surgery related complications. Click here for file
