# Supplementary material for: Clinical outcome of implant removal after fracture healing. Design of a prospective multicentre clinical cohort study
Source: BMC Musculoskelet Disord. 2012 Aug 15;13:147. doi: 10.1186/1471-2474-13-147 (PMC3493388; doi:10.1186/1471-2474-13-147)
Supplement: Additional file 1 — Definitions of surgery related complications. [file 1471-2474-13-147-S1.doc]

**Appendix 1** definitions of surgery related complications

- **Postoperative haemoraghe:** More than normally expected blood loss, this leads to a re-operation or the appearance of a huge hematoma. The incidence in literature is about 1%

## - Wound infection: A strong red colouring of the wound, fluid or pus coming out of the wound or wound distension possible along with a positive wound culture and/or fever. The incidence in literature varies from 1 up to 15%

## - Nerve damage: Injury of a motorised and/or sensible nerve due to the surgery of which the patient derives permanent harm. The incidence in literature varies from 1 up to 29%

## - Refracture: A new fracture across or in the neighbourhood of the formal fracture which seemed to be healed clinically and radio graphically. Refractures appear almost all in the first six months after implant removal. Data about this phenomenon are mostly derived from literature on implant removal in forearm fractures. The incidence varies from 1 up to 30%

## - A poor cosmetic result: A scar that is more unpleasant or ugly after the surgery for implant removal than before this surgery. This subjective parameter can only be assessed by the patient. In literature the incidence is less than 9%
